# Singe intraoperative instillation of chemotherapy during radical cystectomy for bladder cancer: Oncological outcome and survival predictors

**DOI:** 10.1002/cam4.5895

**Published:** 2023-04-16

**Authors:** Jingtian Yang, Kaiwen Li, Yishan Zhang, Jintao Hu, Hao Liu, Wen Dong, Hai Huang, Tianxin Lin, Jian Huang, Wang He

**Affiliations:** ^1^ Department of Urology Sun Yat‐sen Memorial Hospital Sun Yat‐sen University Guangzhou Guangdong P. R. China; ^2^ Guangdong Provincial Key Laboratory of Malignant Tumor Epigenetics and Gene Regulation Sun Yat‐sen Memorial Hospital Sun Yat‐sen University Guangzhou Guangdong P. R. China; ^3^ Guangdong Clinical Research Center for Urological Diseases Guangzhou Guangdong P. R. China

**Keywords:** bladder cancer, disease‐free survival, instillation of chemotherapy, intravesical chemotherapy, overall survival, radical cystectomy

## Abstract

**Purpose:**

To clarify the necessity and effect of a single intraoperative instillation of chemotherapy during radical cystectomy.

**Methods:**

Patients who underwent radical cystectomy for bladder cancer between January 2013 and April 2019 were retrospectively evaluated and divided into a non‐instillation group and an instillation group according to the intraoperative instillation of chemotherapy. Univariate and multivariate Cox regression was used to determine the clinical predictors of overall survival and disease‐free survival. Kaplan–Meier analysis and log‐rank tests were performed to analyze overall survival and disease‐free survival.

**Results:**

Of the 320 patients who were enrolled in the study, 113 underwent radical cystectomy with intraoperative instillation of chemotherapy. Univariate Cox analysis showed that intraoperative instillation was not a risk factor for overall survival or disease‐free survival (HR: 1.04, 95% CI: 0.66–1.63, *p* = 0.864; HR: 1.11, 95% CI: 0.76–1.62, *p* = 0.602, respectively). As shown in the Kaplan–Meier analysis, no significant differences were noted in overall survival (*p* = 0.857) and disease‐free survival (*p* = 0.600) between the two groups. A subgroup analysis demonstrated that intraoperative instillation was not associated with a statistically better overall survival and disease‐free survival in the nonmuscle invasive (*p* = 0.852 and 0.836) and muscle‐invasive (*p* = 0.929 and 0.805) patients.

**Conclusion:**

A single intraoperative instillation of chemotherapy during radical cystectomy was not related to better disease‐free survival or overall survival. It is unnecessary to consider single instillation of chemotherapy as a regular procedure during radical cystectomy.

## Graphical abstract

The effectiveness of intraoperative intravesical chemotherapy is an important, but understudied, for preventing tumor residue. This study aimed to explore and verify the effect of intraoperative instillation of chemotherapy during radical cystectomy. A single intraoperative instillation of chemotherapy during radical cystectomy was not related to better disease free survival or overall survival.

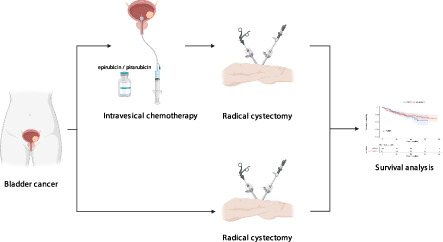

## 1 INTRODUCTION

Bladder cancer is the tenth most common cancer worldwide.^1^ Transurethral resection of bladder tumor (TURBT) with instillation of chemotherapy is the chief treatment for nonmuscle invasive bladder cancer (NMIBC).^2^ The main purpose of instillation of chemotherapy is to reduce recurrence. Previous studies have shown that compared with TURBT alone, the instillation of chemotherapy after TURBT can reduce recurrence in NMIBC patients.^3^, ^4^, ^5^, ^6^, ^7^ Epirubicin and pirarubicin are two of the most effective instillation chemotherapy agents and a single immediate instillation of chemotherapy with epirubicin and pirarubicin can prolong recurrence‐free survival in NMIBC patients.^8^ Furthermore, a single postoperative instillation after TURBT is recommended for NMIBC in the current European Association of Urology guidelines.^9^ In addition, Chang et al. reported that single‐dose instillation of chemotherapy can reduce bladder cancer recurrence in patients who have undergone nephroureterectomy.^10^, ^11^ Urethral cutting is a mandatory operation during RC, and this procedure may lead to tumor cell extravasation. The purpose of instillation is to prevent plant metastasis by destroying free‐floating or superficial cells in the bladder. The implantation of monoclonal, proliferating tumor cells into the urothelium is considered to potentially be the primary cause of recurrence.^11^, ^12^ The effectiveness of intraoperative intravesical chemotherapy is important, but understudied, for preventing tumor residue. It is still unclear whether a single intraoperative instillation of chemotherapy during radical cystectomy (RC) can reduce recurrence and prolong survival, which has never been described. This research provides a timely and necessary study of the effectiveness of intraoperative intravesical chemotherapy.

Therefore, a small number of medical centers have attempted to perform a single instillation of chemotherapy before removing the bladder during RC. The concept of neoadjuvant intravesical chemotherapy before resection of bladder cancer is promising and deserves further evaluation.^13^, ^14^ In phase I clinical study, the safety and efficacy of neoadjuvant intravesical therapy were investigated in patients who were scheduled for RC and not eligible for neoadjuvant chemotherapy.^15^ Within the next few years, perioperative intravesical therapy is set to become a future research priority for the treatment of bladder cancer. As far as we know, there is no relevant research that directly verifies the effect of intraoperative instillation during RC on short‐term or long‐term survival outcomes. This study aimed to explore and verify the effect of intraoperative instillation of chemotherapy during RC.

## 2 METHODS

### 2.1 Patient selection

We retrospectively reviewed all patients diagnosed with bladder cancer who underwent RC from January 2013 to April 2019. We obtained informed consent from the study participants and deidentified all patient details. We have followed the relevant Equator guidelines and the reporting of this study conforms to STROBE guidelines.^16^ The inclusion criteria were as follows: (1) Patients diagnosed with bladder cancer; (2) Patients who underwent RC with or without single intraoperative instillation of chemotherapy; (3) The histologic type was urothelial carcinoma; (4) The demographic, clinical, and pathological information of the patients was exact.

### 2.2 Single instillation of chemotherapy during RC

The single instillation of chemotherapy was defined as performing a single instillation of chemotherapy with epirubicin or pirarubicin before removing the bladder during RC. After surgical area disinfection and catheterization, the bladder was filled with chemotherapy agent solution through a transurethral catheter. Afterward, the chemotherapeutic agents remained in the bladder for approximately 30 minutes. When we were ready to remove the bladder, the chemotherapeutic solution was drained from the bladder through the catheter. Before cutting, we separate and expose the urethra as long as possible, then clamp it with a non‐absorbable polymer clip. In this way, the risk of tumor cell leakage is minimized.

### 2.3 Statistical analysis

The definition of overall survival (OS) was the time from RC to death from any cause; living patients were censored at the time of the last recording. The primary endpoint was disease‐free survival (DFS), which was defined as the time from RC to tumor recurrence (local recurrence or metastatic recurrence) or death from any cause.

The t test and Kruskal‐Wallis test were employed to compare continuous variables. The chi‐square test and Fisher's exact test were used to analyze categorical variables. Univariate and multivariate Cox proportional hazards regression models were used to determine independent risk factors. Kaplan–Meier analysis with the log‐rank test was used to estimate the impact of instillation on OS and DFS. On all tests, a *p* value of <0.05 demonstrated a significant difference, and all tests were two‐sided. Statistical analysis was performed in the “R‐4.0.3‐win” R package.

## 3 RESULTS

A total of 320 patients met the inclusion criteria and were enrolled in the study. The baseline characteristics of the study population are shown in Table 1. In the instillation group, the length of hospital stay was significantly longer (25 vs. 29 days, *p* = 0.001) and more patients underwent neobladder (*p* = 0.017). No significant difference was observed in the other demographic, clinical, and pathological characteristics including age, T stage, N stage, grade, surgical margin, and lymphvascular invasion.

**TABLE 1:** Baseline characteristics of the study population.

Variable	Non‐instillation	Instillation	*p* value
	*n* = 207 (%)	*n* = 113 (%)	
Age, years (mean (SD))	64.08 (10.93)	63.58 (9.35)	0.680
Sex			
Male	175 (84.5)	95 (84.1)	1.000
Female	32 (15.5)	18 (15.9)	
Hospital stays, days (median [IQR])	25.00 [19.00, 32.00]	29.00 [23.00, 35.00]	0.001
BMI			
<18.5	14 (6.8)	7 (6.2)	0.360
18.5–23.9	120 (58.0)	57 (50.4)	
>23.9	73 (35.3)	49 (43.4)	
Operative time, min (mean (SD))	323.25 (90.92)	321.35 (81.00)	0.852
Estimated blood loss, ml (median [IQR])	100.00 [100.00, 200.00]	100.00 [100.00, 150.00]	0.138
Intraoperative blood transfusions	183/23 (88.8/11.2)	104/9 (92.0/8.0)	0.474
ASA			
I	5 (2.4)	0 (0.0)	0.123^a^
II	105 (50.7)	50 (44.2)	
III	92 (44.4)	62 (54.9)	
IV	5 (2.4)	1 (0.9)	
Urinary diversion			
Cutaneous ureterostomy	31 (15.0)	6 (5.3)	0.017
Ileal conduit	25 (12.1)	10 (8.8)	
Neobladder	151 (72.9)	97 (85.8)	
PLND	202 (97.6)	109 (96.5)	0.725^a^
Neoadjuvant chemotherapy	16 (7.7)	8 (7.1)	1.000
T stage			
≤1	68 (32.9)	35 (31.0)	0.786
2	74 (35.7)	40 (35.4)	
3	35 (16.9)	24 (21.2)	
4	30 (14.5)	14 (12.4)	
N stage			
N0	163 (78.7)	80 (70.8)	0.146
N+	44 (21.3)	33 (29.2)	
Grade			
High grade	194 (93.7)	106 (93.8)	1.000^a^
Low grade	13 (6.3)	7 (6.2)	
Positive surgical margin	15 (7.2)	13 (11.5)	0.280
Positive LVI	47 (22.7)	36 (31.9)	0.099

Abbreviations: ASA, American Society of Anesthesiologists; BMI, body mass index; LVI: lymphvascular invasion; PLND, pelvic lymph node dissection.

^a^ Fisher's exact test.

**FIGURE 1:**
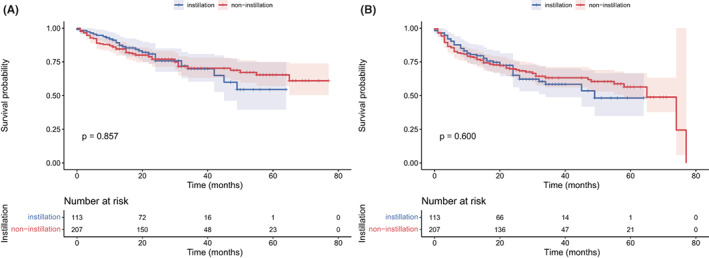
Kaplan–Meier curve of overall survival (A) and disease‐free survival (B) according to intraoperative instillation.

**FIGURE 2:**
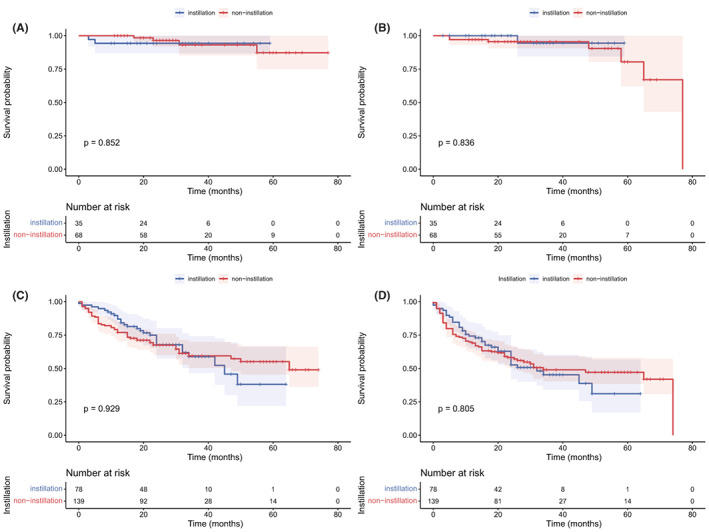
Kaplan–Meier curve of overall survival and disease‐free survival in nonmuscle invasive (A, B) and muscle‐invasive (C, D) bladder cancer according to intraoperative instillation.

**FIGURE 3:**
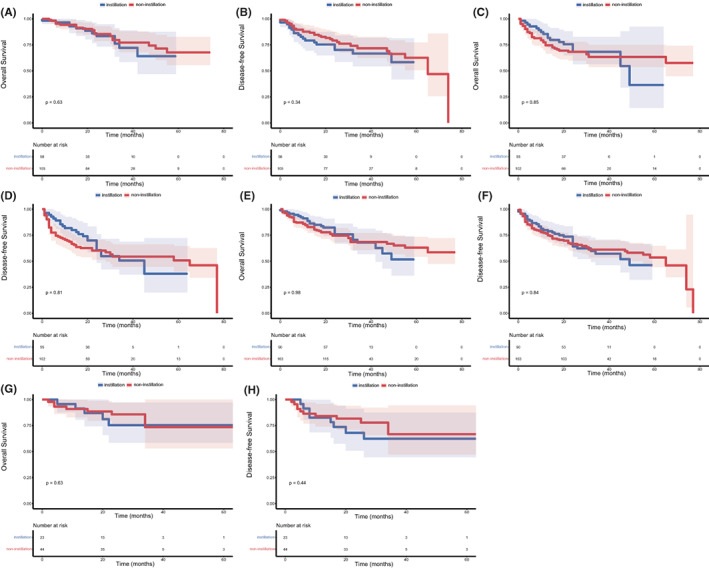
Subgroup analyses of Kaplan–Meier curves according to tumor size (≤3 cm: A and B; > 3 cm: C and D) and tumor number (single: E and F; multiple:G and H).

The median follow‐up was 28 months. As shown in Figure 1, no significant difference was observed in OS (*p* = 0.857) and DFS (*p* = 0.600) between the non‐instillation and instillation groups. The difference in OS and DFS remained not statistically significant in the NMIBC (*p* = 0.852 and 0.836; Figure 2A,B) patients and MIBC (*p* = 0.929 and 0.805; Figure 2C,D) patients. Subgroup analyses based on tumor size and tumor number were performed and are presented in Figure 3. The difference in OS and DFS remained not statistically significant in each subgroup (Figure 3, all *p* > 0.05). In Figure 1 and Figure 2, it can be seen that no cumulative probability of survival was assigned to the last patient of the non‐instillation group because of the event of DFS (the cumulative probability was 0/1), and there is a sudden drop to 0 in the probability for this reason.

The univariate and multivariate Cox regression outcomes are listed in Tables 2 and 3. The results demonstrated that instillation was not a risk factor for either DFS or OS (HR: 1.04, 95% CI: 0.66–1.63, *p* = 0.864; HR: 1.11, 95% CI: 0.76–1.62, *p* = 0.602, respectively). In addition, we found that age, urinary diversion, T stage and N stage were independent risk factors for both DFS and OS. Increasing age was related to decreased OS (HR: 1.04, CI:1.02–1.07, *p* < 0.001) and DFS (HR: 1.03, CI:1.01–105, *p* = 0.005). Compared to cutaneous ureterostomy, neobladder had an improved OS (HR: 0.45, CI: 0.26–0.80, *p* = 0.006) and DFS (HR: 49, CI: 0.30–0.82, *p* = 0.007). For pathological outcomes, T stage and N stage were associated with decreased OS and DFS (both *p* < 0.001).

**TABLE 2:** Prognostic factors for overall survival of patients.

Variable	Univariate analysis			Multivariate analysis		
	HR	95% CI	*p* value	HR	95% CI	*p* value
Instillation						
Non‐instillation	Reference			—	—	—
Instillation	1.04	0.66–1.63	0.864	—	—	—
Age	1.04	1.02–1.07	<0.001	1.04	1.02–1.07	<0.001
Sex						
Male	Reference			—	—	—
Female	1.30	0.76–2.24	0.339	—	—	—
Hospital stays	0.98	0.96–1.00	0.119	—	—	—
BMI						
<18.5	Reference			—	—	—
18.5–23.9	0.84	0.40–1.75	0.635	—	—	—
>23.9	0.45	0.20–1.02	0.057	—	—	—
Operative time	1.00	1.00–1.00	0.362	—	—	—
Estimated blood loss	1.00	1.00–1.00	0.232	—	—	—
Intraoperative blood transfusions						
No	Reference			—	—	—
Yes	1.29	0.68–2.44	0.433	—	—	—
ASA						
I	Reference			—	—	—
II	0.77	0.11–5.67	0.799	—	—	—
III	1.38	0.19–9.98	0.753	—	—	—
IV	1.78	0.16–19.73	0.637	—	—	—
Urinary diversion						
Cutaneous ureterostomy	Reference			Reference		
Ileal conduit	1.05	0.56–1.96	0.879	0.92	0.46–1.83	0.805
Neobladder	0.28	0.17–0.48	<0.001	0.45	0.26–0.80	0.006
PLND						
No	Reference			—	—	—
Yes	1.47	0.36–5.96	0.593	—	—	—
Neoadjuvant chemotherapy						
No	Reference			—	—	—
Yes	1.17	0.54–2.54	0.693	—	—	—
T stage						
≤1	Reference			Reference		
2	4.48	1.85–10.86	0.001	3.86	1.57–9.49	0.003
3	11.64	4.84–28.00	<0.001	6.86	2.72–17.31	<0.001
4	13.38	5.43–32.96	<0.001	5.77	2.18–15.30	<0.001
N stage						
N0	Reference			Reference		
N+	4.89	3.19–7.48	<0.001	2.83	1.62–4.95	<0.001
Grade						
High grade	Reference			—	—	—
Low grade	0.00	0–Inf	0.995	—	—	—
Surgical margin						
Negative	Reference			Reference		
Positive	1.92	1.07–3.47	0.030	1.15	0.61–2.18	0.664
LVI						
Negative	Reference			Reference		
Positive	2.92	1.9–4.48	<0.001	1.23	0.71–2.13	0.458

Abbreviations: ASA, American Society of Anesthesiologists; BMI, body mass index; CI, confidence interval; HR, hazard ratio; LVI: lymphvascular invasion; PLND, pelvic lymph node dissection.

**TABLE 3:** Prognostic factors for disease‐free survival of patients.

Variable	Univariate analysis			Multivariate analysis		
	HR	95% CI	*p* value	HR	95% CI	*p* value
Instillation						
Non‐instillation	Reference			—	—	—
Instillation	1.11	0.76–1.62	0.602	—	—	—
Age	1.03	1.01–1.05	0.001	1.03	1.01–1.05	0.005
Sex						
Male	Reference			—	—	—
Female	1.25	0.77–2.03	0.357	—	—	—
Hospital stays	0.98	0.96–1.00	0.046	0.99	0.97–1.01	0.289
BMI						
<18.5	Reference			—	—	—
18.5–23.9	0.85	0.44–1.66	0.640	—	—	—
>23.9	0.55	0.27–1.11	0.097	—	—	—
Operative time	1.00	1.00–1.00	0.480	—	—	—
Estimated blood loss	1.00	1.00–1.00	0.310	—	—	—
Intraoperative blood transfusions						
No	Reference			—	—	—
Yes	1.05	0.58–1.92	0.865	—	—	—
ASA						
I	Reference			—	—	—
II	1.15	0.16–8.36	0.889	—	—	—
III	1.85	0.26–13.38	0.54	—	—	—
IV	1.71	0.16–18.91	0.661	—	—	—
Urinary diversion						
Cutaneous ureterostomy	Reference			Reference		
Ileal conduit	0.79	0.44–1.41	0.421	0.68	0.36–1.31	0.252
Neobladder	0.29	0.19–0.46	<0.001	0.49	0.30–0.82	0.007
PLND						
No	Reference			—	—	—
Yes	2.06	0.51–8.35	0.310	—	—	—
Neoadjuvant chemotherapy						
No	Reference			—	—	—
Yes	0.95	0.46–1.95	0.885	—	—	—
T stage						
≤1	Reference			Reference		
2	3.67	1.88–7.14	<0.001	3.16	1.59–6.26	0.001
3	8.36	4.24–16.5	<0.001	5.28	2.54–10.95	<0.001
4	10.90	5.46–21.77	<0.001	5.70	2.65–12.23	<0.001
N stage						
N0	Reference			Reference		
N+	4.00	2.77–5.79	<0.001	2.65	1.64–4.28	<0.001
Grade						
High grade	Reference			Reference		
Low grade	0.21	0.05–0.85	0.029	0.62	0.15–2.63	0.515
Surgical margin						
Negative	Reference			—	—	—
Positive	1.57	0.91–2.71	0.107	—	—	—
LVI						
Negative	Reference			Reference		
Positive	2.47	1.7–3.6	<0.001	1.03	0.64–1.64	0.911

Abbreviations: ASA, American Society of Anesthesiologists; BMI, body mass index; CI, confidence interval; HR, hazard ratio; LVI: lymphvascular invasion; PLND, pelvic lymph node dissection.

## 4 DISCUSSION

Surgical intervention is a pivotal medical decision for bladder cancer patients. RC with standard lymph node dissection is still the primary treatment for high‐risk NMIBC and MIBC. Among patients with NMIBC, only those who are at the highest risk of disease progression (Subgroup of highest‐risk tumors in risk stratification, such as recurrence or failure after intravesical BCG immunotherapy and repeated recurrent T1HG) are considered for RC. In our institution, the potential benefit must be weighed against its risks, morbidity, and impact on quality of life and discussed with patients, in a shared decision‐making process. All NMIBC patients in this study have experienced the multi‐disciplinary treatment (MDT) discussion and decision‐making process before RC. It has been reported that RC can effectively control local recurrence with a five‐year DFS of 74%. For NMIBC patients, the five‐year DFS after RC was 94%.^17^ However, a few studies also reported that nearly half of the patients progressed to local or distant metastasis within approximately 2 to 3 years after RC.^18^, ^19^, ^20^, ^21^, ^22^

The purpose of instillation is to prevent implantation metastasis during RC by killing free‐floating or superficial tumor cells in the bladder. Nonetheless, no presented evidence or research has indicated that instillation during RC can effectively reduce recurrence or improve survival. Our study aims to explore and verify the validity of intraoperative instillation during RC.

It has been demonstrated that epirubicin and pirarubicin are equally effective in preventing tumor recurrence.^23^, ^24^ Moreover, both epirubicin and pirarubicin are anthracyclines and have similar pharmacological effects.^25^ In our study, we found that instillation was also not a risk factor for either DFS or CSS. Moreover, instillation was not statistically associated with either DFS or OS in the Kaplan–Meier analysis. Thus, our results showed that instillation could not decrease the risk of recurrence after RC or improve the survival rate. Consistent with earlier results, we also found that age, T stage, and N stage were independent predictors of survival in bladder cancer, which has been demonstrated by previous studies.^26^, ^27^

In this study, our results indicate for the first time that instillation during RC did not reduce recurrence or prolong survival for bladder cancer. Although immediate instillation of chemotherapy after TURBT has been shown to reduce recurrence, the validity of instillation during RC for patients receiving RC is still unknown and has never been reported in the previous literature. There is no reliable evidence for this practice. Therefore, our study provided clear evidence of the effectiveness of instillation during RC and may help guide clinicians to make treatment decisions for patients with bladder cancer. In other words, instillation cannot be an effective measure to reduce recurrence and improve survival and should not be recommended.

Our results could be explained by several reasons. In previous studies, a single instillation after TURBT can reduce the recurrence of NMIBC,^3^, ^4^, ^5^, ^6^, ^7^ but for high‐risk NMIBC and MIBC, its effectiveness is questionable. Free‐floating or superficial tumor cells in the bladder can be killed by chemotherapeutics, but MIBC has a deeper invasion and a higher possibility of micrometastasis. Furthermore, the effectiveness of instillation is limited. The concentration of the chemotherapeutic agent is affected by continuous urine secretion from the kidneys, which limits the concentration of the drug and reduces the efficacy. In addition, the urothelium acts as a barrier, preventing chemotherapeutics from reaching the deeper muscle layer of the bladder.^28^

More significance should be placed on intraoperative tumor‐free procedures than on the intraoperative instillation of chemotherapy. One of the most important objectives of tumor‐free procedures is to avoid positive surgical margins. Positive surgical margins after RC result in poorer prognosis for patients with bladder cancer. Half of them will recur within a year and die of bladder cancer.^29^ Different locations of positive surgical margins represent distinct risk factors. Urethral and soft‐tissue positive surgical margins were correlated with worse disease‐specific survival.^30^ To achieve the reduction of the primary tumor, pathological staging, and local cancer control, neoadjuvant therapy should be considered to reduce the potential of soft‐tissue positive margins.^30^, ^31^ Negative frozen section analysis or a negative preoperative endoscopic urethral sampling prior to the RC was recommended to reduce the risk of urethral‐positive surgical margins.^32^ There are many drawbacks of instillation during RC. First, the safety and side effects cannot be ignored. The toxicity of chemotherapeutic agents may lead to local and systemic side effects. The most common local side effect is chemical cystitis, which can induce a series of symptoms including dysuria, frequency, and urgency, gross hematuria. The possibility of perforation also exists, which may cause bladder necrosis or peritonitis.^33^ Although this is a rare complication, it has been reported. Systemic effects are rare and primarily result in myelosuppression with rates of approximately 1%.^5^, ^28^, ^34^, ^35^ Second, performing instillation during RC increases the workload of the medical staff and prolongs the preparation time of surgery for each patient. Moreover, it increases the medical expense of patients. In addition, chemotherapeutic agents can enter the body in a low dose through skin contact or inhalation over a long period of time, posing a threat to the health of medical staff.

In summary, our study attempts to notify clinicians that it is unreasonable to perform instillation during RC, and not using this procedure can not only prevent relative side effects caused by intravesical therapy but also reduce the financial burden of patients.

There are some limitations of the present study. First, it was a retrospective study and bias within the process of collecting information was inevitable. Second, the number of patients was relatively small. Third, the calculation and justification of the sample size selected for this study were not performed because we enrolled all patients who met the inclusion criteria to obtain larger cohorts. Therefore, our study is limited by a small single‐center cohort and the study's retrospective design. Therefore, a long‐term, multicenter, and large‐scale prospective study will be needed. To solve the problems above, the improvement measure is performing long‐term, multicenter, and large‐scale prospective studies.

## 5 CONCLUSION

A single intraoperative instillation of chemotherapy during RC cannot provide improved disease‐free survival and overall survival. The use of single instillation of chemotherapy during radical cystectomy does not reduce the risk of recurrence or improve survival. It is unreasonable to consider a single instillation of chemotherapy as a regular procedure during radical cystectomy.

## AUTHOR CONTRIBUTIONS

**Jingtian Yang, Kaiwen Li and Yishan Zhang:** Conceptualization, data curation, project administration and writing – original draft. **Jintao Hu:** Data curation, formal analysis and software. **Hao Liu:** Supervision. **Wen Dong:** Data curation and formal analysis. **Hai Huang:** Data curation and methodology. **Tianxin Lin, Jian Huang and Wang He:** Conceptualization, supervision, visualization, writing – review and editing.

## FUNDING INFORMATION

This study was funded by the National Key Research and Development Program of China (Grant No. 2018YFA0902803, 2017YFC1308600); the National Natural Science Foundation of China (Grant No. 81825016, 81,802,530, 81,830,082, 81,672,395, 81,871,945, 81,772,719, 81,772,728, 2,072,639, 91,740,119, 81,472,381, 81,972,385, 82,173,266); the Key Areas Research and Development Program of Guangdong (Grant No. 2020A1515010815, 2018B010109006, 2017A020215072); the Science and Technology Planning Project of Guangdong Province (Grant No. 202002030388, 201,803,010,049, 2017B020227007, 201,704,020,097); Guangdong Clinical Research Center for Urological Diseases (Grant No. 2020B1111170006); Yixian Youth project of Sun Yat‐sen Memorial Hospital (Grant No. YXQH201812); Young Teacher Training Funding of Sun Yat‐sen University (Grant No. 19ykzd21, 19ykpy121).

## CONFLICT OF INTEREST STATEMENT

The authors have no relevant financial or non‐financial interests to disclose.

## ETHICS STATEMENT

The study was an observational retrospective study and was approved by the ethics committee of Sun Yat‐sen Memorial Hospital (Approval No. SYSEC‐KY‐KS‐2021‐135). All procedures performed in studies involving human participants were under the ethical standards of the institutional research committee and with the 1964 Helsinki declaration and its later amendments or comparable ethical standards. We have received broad consent from the ethics committee of Sun Yat‐sen Memorial Hospital.

## CLINICAL TRIAL REGISTRATION

The trial registration number: ChiCTR2100048974 (Chinese Clinical Trial Registry).

Date of registration: 2021/07/19.

## Data Availability

The data that support the findings of this study are available from the corresponding author, Wang He, upon reasonable request. All authors agree to the journal to review the data if needed.
